# Adenovirus E4ORF1 activates isoform-specific phosphatidylinositol 3-kinase signaling in human endothelial cells

**DOI:** 10.1016/j.jbc.2025.110947

**Published:** 2025-11-13

**Authors:** Fuqiang Geng, Mariko Kobayashi, Yang Lin, Jesus Maria Gomez-Salinero, Dominick Romano, Jean Kanyo, Jennifer Geng, Ying Liu, Michael Ginsberg, Jae-Hung Shieh, Kevin Chen, TuKiet T. Lam, Arash Rafii, Sina Y. Rabbany, Raphaël Lis, Shahin Rafii

**Affiliations:** 1 Division of Regenerative Medicine, Hartman Institute for Therapeutic Organ Regeneration, Department of Medicine, Ansary Stem Cell Institute, Weill Cornell Medicine, New York, New York, USA; 2 DeMatteis School of Engineering and Applied Science, Hofstra University, Hempstead, New York, USA; 3 MS & Proteomics Resource of the W.M. Keck Biotechnology Resource Laboratory, Yale University School of Medicine, New Haven, Connecticut, USA; 4 Department of Molecular Biophysics & Biochemistry, Yale University, New Haven, Connecticut, USA; 5 Angiocrine Bioscience, Inc., San Diego, California, USA; 6 Stem Cell and Microenvironment Laboratory, Weill Cornell Medicine-Qatar, Doha, Qatar

**Keywords:** adenovirus, AKT, angiogenesis, DLG1, E4ORF1, endothelium, phosphatidylinositol-3-kinase

## Abstract

The human adenovirus serotype 5 E4ORF1 (Ad5E4ORF1) protein promotes primary endothelial cell survival and angiocrine functions by hijacking the cellular phosphatidylinositol 3-kinase (PI3K)/AKT signaling pathway. However, the mechanism by which E4ORF1 activates PI3K in vascular cells remains largely unknown. Here, we show that Ad5E4ORF1 recruits multiple host scaffold proteins, including DLG1, which facilitates AKT activation in response to both Ad5E4ORF1 and endogenous receptor agonists in human endothelial cells. Furthermore, Ad5E4ORF1 specifically engages the human PI3K isoform p110α-p85β through multidomain interactions exclusively with p110α. Notably, E4ORF1 proteins from different adenoviral serotypes differentially interact with p110α, resulting in varying levels of AKT activation in endothelial cells. We propose that E4ORF1 specifically recognizes and allosterically activates p110α-p85β *via* direct multisite contacts with p110α.

Enforced expression of human adenovirus serotype 5 early gene E4ORF1 (Ad5E4ORF1) in vascular endothelial cells preserves their cellular identity *in vitro* and sustains their angiogenic and angiocrine functions. Importantly, Ad5E4ORF1 augments the survival of mouse and human adult endothelial cells even in the absence of serum and growth factors (1, 2, 3), enabling E4ORF1-transduced endothelial cells to function as a robust supportive niche for stem cells (4). The independence of these cells from xenobiotic and supplemental factors allows precise interrogation of niche-stem cell cross-signaling without confounding external influences (5). One signaling pathway activated by Ad5E4ORF1 and several other E4ORF1 serotypes is the phosphatidylinositol 3-kinase (PI3K)/AKT axis (2, 4). For instance, Ad9E4ORF1 stimulates PI3K signaling to support anchorage-independent growth in adherent cells (6), while Ad36E4ORF1 enhances cellular glucose uptake independently of insulin (7, 8) and improves glycemic control in mice (9, 10). Similarly, Ad5E4ORF1 increases glucose uptake in adipose cells through both insulin-dependent and insulin-independent pathways (11). In addition, Ad5E4ORF1 is essential for efficient viral replication in primary cells (12) and exhibits immunosuppressive activity (13).

Growth factors and chemokines activate class I PI3K through cell-surface receptor tyrosine kinases (RTKs) and G protein–coupled receptors (GPCRs). Upon receptor activation, PI3K is recruited to the plasma membrane, where its intramolecular self-inhibition is relieved (14). Activated PI3K then converts phosphatidylinositol 4,5-bisphosphate (PIP2) to phosphatidylinositol 3,4,5-trisphosphate (PIP3), which serves as a docking site for AKT and other downstream targets such as Ras-related C3 botulinum toxin substrate 1 (Rac1) and serum and glucocorticoid-inducible kinase (SGK) (15). Full activation of AKT involves PIP3-mediated relief of self-inhibition and subsequent sequential phosphorylation of T308 by PDK1 and S473 by mTORC2. Activated AKT then phosphorylates a host of downstream substrates to promote cell survival, proliferation, growth, and metabolic reprogramming (16). The specific signaling output downstream of PI3K is ultimately determined by the particular PI3K isoform(s) engaged, which are differentially expressed across tissues and uniquely adapted to integrate distinct extracellular cues (17, 18).

E4ORF1 activation of PI3K is evidenced by increased cellular PI3K catalytic activity and the accumulation of PI3K and PIP3 at the plasma membrane in E4ORF1-expressing cells (11, 19). Earlier studies, primarily using immortalized cell lines and focusing on Ad9E4ORF1-mediated PI3K signaling, identified critical E4ORF1 domains required for PI3K activation. These include the C-terminal PDZ-binding motif (PBM) and an ill-defined vesicle-targeting region known as M2 (20, 21, 22). Ad9E4ORF1 forms homotrimers in the cell, and the trimeric PBMs cooperatively and selectively bind the tandem PDZ1 and PDZ2 domains of discs large homolog 1 (DLG1), a key interaction necessary for Ad9E4ORF1-mediated AKT activation (23, 24). More recent investigations revealed a conserved, direct interaction between PI3K and multiple E4ORF1 proteins, including Ad5E4ORF1, leading to the proposal that membrane-localized DLG1 (25, 26), *via* its interaction with E4ORF1, recruits PI3K to the plasma membrane (27, 28). However, the precise PI3K form(s) involved and the mechanism underlying the activation of catalytically inhibited PI3K remain unclear.

In addition to activating PI3K, E4ORF1 proteins have also been shown to activate rat sarcoma (RAS) (29) and extracellular signal-regulated kinase (ERK) (30) under certain experiment conditions. Moreover, Ad5E4ORF1 enhances cellular glucose anabolism through physical interaction with nuclear Myc (31). To understand how Ad5E4ORF1-engineered endothelial cells sustain their proangiogenic and pro-stem cell function, we sought to identify Ad5E4ORF1-interacting proteins in primary human endothelial cells. Here, we characterized previously unrecognized interactions between E4ORF1 and host signaling proteins and provide evidence supporting a novel mechanism of PI3K activation.

## Results

### Functional N terminally tagged Ad5E4ORF1 proteins

**Figure 1:**
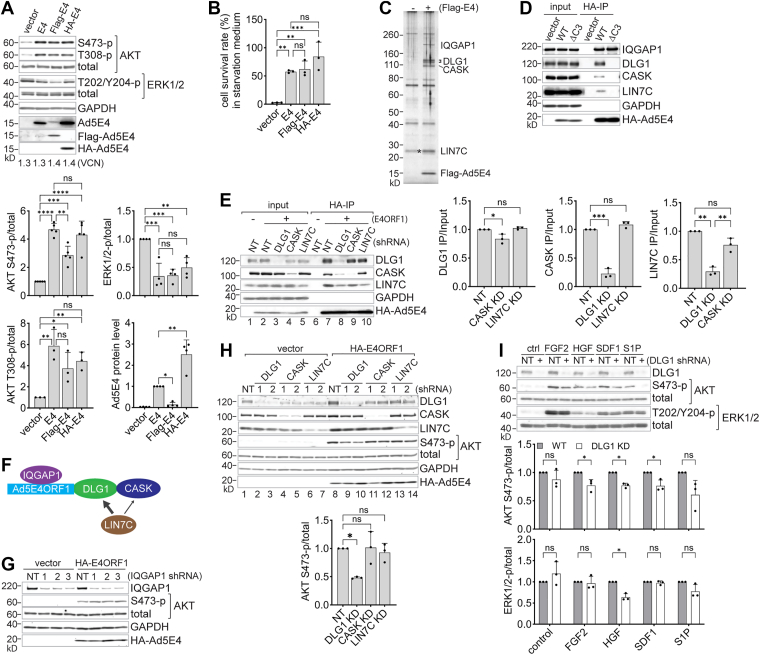
**Human Ad5E4ORF1 associates with scaffold proteins IQGAP1, DLG1, CASK, and LIN7C but only DLG1 promotes E4ORF1-mediated AKT activation**. *A*, western blot analysis of Ad5E4ORF1 proteins and phosphorylated AKT and ERK1/2 in HUVECs. (*Top panel*) Cells infected with vector control or Ad5E4ORF1 viruses were starved for 4 h prior to cell lysis in LDS loading buffer. Tagged and untagged Ad5E4ORF1 proteins were probed with Ad5E4ORF1-, Flag-, or HA-specific antibodies. VCN (*bottom*), genomic lentiviral vector copy numbers. (*Lower panels*) Densitometry quantification of immunoblots shown in the *top panel*. Phosphorylated AKT and ERK signals were normalized to their respective total protein levels and expressed relative to the vector control, which was set to 1. Ad5E4ORF1 protein levels, normalized to GAPDH, were quantified from anti-Ad5E4ORF1 immunoblots and expressed relative to native (untagged) E4ORF1. Data are presented as mean ± SD from 3 to 5 independent HUVEC lines. Statistical analysis was performed using one-way ANOVA (two-sided), followed by Tukey’s *post hoc* test. ns, nonsignificant; ∗, *p* < 0.05; ∗∗, *p* < 0.01; ∗∗∗, *p* < 0.001; ∗∗∗∗, *p* < 0.0001 (*p* value notation used throughout all figures). *B*, Ad5E4ORF1-enabled cell survival under starvation. Indicated transduced HUVECs were kept in minimal X-Vivo 20 medium for 7 days and cell survival rates (percentage of live cells in starvation relative to those in growth medium) presented as mean ± SD. One-way ANOVA with Tukey’s test (n = 3 independent HUVEC lines). *C*, identification of Ad5E4ORF1-interacting proteins in HUVECs. Cells expressing GFP (−) or Flag-Ad5E4ORF1 (+) were subjected to cross-linking anti-Flag immunoprecipitation. Flag peptide-eluted proteins were resolved on SDS-PAGE and gel slices analyzed by mass spectrometric protein ID. Shown is an SDS-PAGE silver stain with major identified candidate proteins marked on the right. The band corresponding to LIN7C was identified based on increased signal intensity over a comigrating background protein, denoted by an *asterisk* (∗). *D*, confirmation of identified interactions by protein immunoprecipitation (IP) in HUVECs expressing HA-tagged wild type (WT) or mutant Ad5E4ORF1. ΔC3, deletion of C terminal 3 amino acids (part of PDZ-binding motif). Whole cell lysates were prepared in IGEPAL CA-630 buffer without cell cross-linking. Bead-bound proteins, along with 15% of the corresponding input lysates, were analyzed by western blot. Data shown represent experiments using three HUVEC lines. *E*, interdependence of Ad5E4ORF1 scaffold interactions. DLG1, CASK, or LIN7C gene-specific shRNAs or a nontargeting (NT) shRNA were stably expressed under doxycycline induction for 4 days in control and HA-Ad5E4ORF1-transduced HUVECs. (*Left panel*) Immunoprecipitation blot performed as in (*D*). (*Right panels*) Densitometry quantification of DLG1, CASK, and LIN7C IP efficiency (IP/Input) in gene knockdown (KD) HUVECs relative to NT control. One-way ANOVA with Tukey’s test (mean ± SD; n = 3 HUVEC lines). *F*, schematic model of Ad5E4ORF1 interactions with scaffold proteins. *G* and *H*, effect of scaffold protein knockdowns on AKT activation. Indicated gene-specific shRNAs were doxycycline-induced for 4 days in HUVECs, which were then starved for 4 h and subjected to western blot analysis. Data shown represent experiments using three HUVEC lines. (*H*, *lower panel*) Quantification of AKT phosphorylation (average of two shRNAs) from *upper panel* blots. One-way ANOVA with Tukey’s test (mean ± SD; n = 3 HUVEC lines). *I*, effect of DLG1 knockdown on receptor-mediated AKT activation. DLG1-specific (+) and nontargeting (NT) shRNAs were doxycycline-induced in HUVECs for 4 days. After 4 h of starvation, cells were stimulated for 5 min with the indicated cytokines (20 ng/ml FGF2, HGF, SDF1; or 100 nM S1P) and lysed directly into LDS loading buffer for western blot. (*Lower panels*) Quantification of AKT and ERK phosphorylation from *upper panel* blots. Paired *t* test (mean ± SD; n = 3 HUVEC lines). Ad5E4ORF1, human adenovirus serotype 5 early gene E4ORF1; AKT, protein kinase B; CASK, calcium/calmodulin-dependent serine protein kinase; DLG1, discs large homolg 1; ERK, extracellular signal-regulated kinase; FGF2, fibroblast growth factor 2; HGF, hepatocyte growth factor; HUVEC, human umbilical vein endothelial cells; IQGAP1, IQ motif containing GTPase activating protein 1; LDS, lithium dodecyl sulfate; LIN7C, Lin-7 Cell Polarity Scaffold C; SDF1, stromal cell-derived factor-1; VCN, viral copy number.

To identify cellular proteins targeted by Ad5E4ORF1 in primary human endothelial cells, we employed a protein immunoprecipitation (IP)-mass spectrometry approach. Due to the lack of an immunoprecipitation-compatible antibody against Ad5E4ORF1, we epitope-tagged E4ORF1 at its N terminus with Flag or hemagglutinin (HA). To assess if epitope tagging affected E4ORF1 function, we used lentiviral vectors to express both tagged and untagged (native) E4ORF1 in human umbilical vein endothelial cells (HUVECs) and compared their ability to activate AKT by western blot analysis and densitometric quantification (Fig. 1*A*).

Despite comparable levels of viral infection, as indicated by similar integrated viral copy numbers (VCNs; bottom of top panel), western blotting using antibodies against native Ad5E4ORF1 revealed differential protein expression depending on the tag. HA-E4ORF1 was expressed at higher levels than the native E4ORF1, whereas Flag-E4ORF1 was expressed at much lower levels (Fig. 1*A*, *bottom right graph*). Nevertheless, all three E4ORF1 versions elicited similar levels of AKT phosphorylation at S473 and T308 (Fig. 1*A*, *left graphs*), indicating that a limiting host factor is likely saturated at low E4ORF1 protein levels. The modest reduction in AKT S473 phosphorylation in Flag-E4ORF1–expressing cells correlates with its lower expression.

As previously reported (3), Ad5E4ORF1 does not induce ERK1/2 activation in HUVECs (Fig. 1*A*, *upper right graph*). The modest reduction in ERK1/2 phosphorylation observed in E4ORF1-expresssing cells is likely due to negative feedback from sustained PI3K-AKT signaling. Importantly, all three versions of Ad5E4ORF1 supported cell survival equally well in growth factor-free conditions (Fig. 1*B*).

In summary, although the Flag and HA tags appear to influence Ad5E4ORF1 interactions and potentially affect some of its functions, both tagged versions remain functionally intact with respect to AKT activation and support of cell survival.

### Identification of Ad5E4ORF1-associated proteins in primary human cells

We then chose Flag-E4ORF1 for immunoprecipitation to take advantage of the robust Flag-tag binding and elution system and the low protein expression of Flag-E4ORF1, which may enhance its incorporation into protein complexes. We applied a protein cross-linker prior to cell lysis to preserve native protein interactions and to enrich E4ORF1-proximal proteins. GFP-expressing HUVECs were processed in parallel as a negative control. Staining of immunoprecipitated proteins resolved by SDS-PAGE revealed several prominent bands specific to the Flag-E4ORF1 pull-down, in addition to the expected 15-kDa Flag-E4ORF1 band (Fig. 1*C*). For mass spectrometry analysis, immunoprecipitated proteins from both Flag-E4ORF1-expressing and GFP control cells were briefly resolved by SDS-PAGE followed by colloidal blue staining. The corresponding gel lanes were excised into comparable slices, digested in-gel with trypsin, and analyzed by mass spectrometry. Fifty proteins with two or more unique peptides were specifically identified in the Flag-E4ORF1 samples (Table S1). Based on the abundance of recovered peptides and the corresponding protein sizes we were able to identify the most prominent protein bands as scaffold proteins IQ motif containing GTPase activating protein 1 (IQGAP1), DLG1, calcium/calmodulin-dependent serine protein kinase (CASK), and Lin-7 Cell Polarity Scaffold C (LIN7C) (Fig. 1*C*).

Of the putative Ad5E4ORF1-interacting proteins identified, only DLG1 is known to bind E4ORF1 *via* its PDZ domains and E4ORF1’s C-terminal PBM. We next sought to characterize the newly identified E4ORF1 interactions in HUVECs by immunoprecipitation followed by immunoblotting using protein-specific antibodies (Fig. 1*D*). We also expressed an Ad5E4ORF1 mutant lacking the last three C-terminal amino acids (ΔC3), rendering it deficient in PDZ-binding.

All four scaffold proteins were detected in anti-HA immunoprecipitates from HUVECs expressing wild type (WT) HA-E4ORF1, but not in the lentiviral vector control. Furthermore, while IQGAP1 coprecipitated with both WT and ΔC3 E4ORF1 at comparable levels, DLG1, CASK, and LIN7Cs failed to co-immunoprecipitate with the ΔC3 mutant. These findings demonstrate that Ad5E4ORF1 interacts with DLG1, CASK, and LIN7C through its PDZ-binding motif, whereas its interaction with IQGAP1 occurs through a PDZ-binding motif-independent mechanism.

DLG1, CASK, and LIN7C each contain one or more PDZ domains and may independently bind to E4ORF1. However, given that CASK can complex with either DLG1 (32) or LIN7C (33) in mammals, and that a DLG1-CASK-LIN7C tripartite complex has been identified in fly (34), it is also plausible that these proteins associate with E4ORF1 as a preformed complex. To distinguish these possibilities, we depleted each of them in HUVECs by doxycycline-induced shRNA knockdowns and assessed their interaction with Ad5E4ORF1 by co-immunoprecipitation (co-IP) followed by quantification of each protein’s co-IP efficiency with Ad5E4ORF1 (Fig. 1*E*).

We found that while DLG1 co-IP with Ad5E4ORF1 was only modestly reduced upon CASK knockdown (left graph), both CASK and LIN7C co-IP with Ad5E4ORF1 was markedly reduced following DLG1 knockdown (right graphs). In addition, LIN7C co-IP with Ad5E4ORF1 was substantially and consistently reduced upon CASK knockdown, although the reduction is not statistically significant. These results demonstrate that DLG1 plays a central role in mediating the association of CASK and LIN7C with Ad5E4ORF1 (Fig. 1*F*). Moreover, they suggest that LIN7C can interact with DLG1 independently of CASK.

### DLG1 promotes both Ad5E4ORF1- and receptor-mediated AKT activation

Given the previously reported critical role of DLG1 in E4ORF1-mediated AKT activation, we next sought to determine whether this activity is conserved in HUVECs and to additionally examine the roles of IQGAP1, CASK, and LIN7C. Using shRNA-mediated protein knockdown, we found that depletion of DLG1, but not of CASK, LIN7C (Fig. 1*H*), or IQGAP1 (Fig. 1*G*), significantly reduced AKT S473 phosphorylation in Ad5E4ORF1-transduced HUVECs, suggesting that DLG1 promotes Ad5E4ORF1-mediated AKT activation and acts independently of its associated CASK and LIN7C.

DLG1 can either promote (28, 35, 36) or inhibit (28, 37, 38) PI3K/AKT signaling depending on cellular context. To assess its broader role in signaling, we examined the effect of DLG1 knockdown on cytokine-stimulated AKT activation in naïve HUVECs. Cells were stimulated with tyrosine kinase receptor agonists fibroblast growth factor 2 (FGF2) and hepatocyte growth factor (HGF), as well as G protein–coupled receptors agonists stromal cell-derived factor-1 (SDF1, CXCL12) and sphingosine 1-phosphate (S1P). As shown in Figure 1*I*, DLG1 depletion significantly impaired AKT S473 phosphorylation in response to FGF2, HGF, and SDF1, but had no statistically significant effect on S1P-induced AKT activation (upper graph). In terms of ERK1/2 activation, DLG1 depletion only significantly reduced HGF-stimulated ERK1/2 phosphorylation (lower graph).

Together, these results suggest that DLG1, a facilitator of E4ORF1 activity, functions broadly, if not universally, as a positive regulator of receptor-mediated AKT activation in HUVECs.

### Widespread subcellular distribution of Ad5E4ORF1

**Figure 2:**
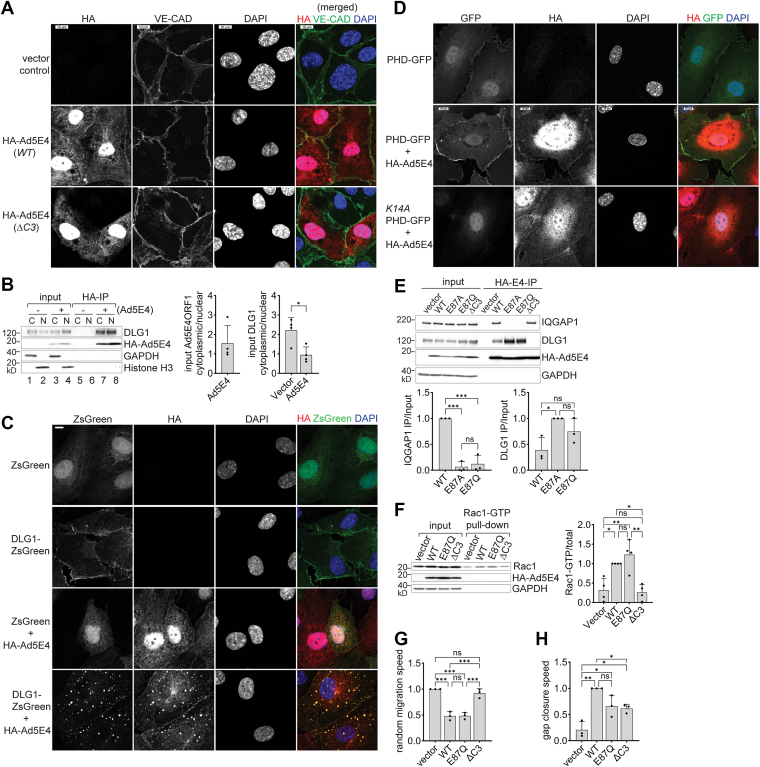
**Ad5E4ORF1 activates PI3K at the plasma membrane**. *A*, subcellular localization of Ad5E4ORF1 *via* indirect immunostaining and confocal fluorescence microscopy. HUVECs (vector control or expressing HA-tagged WT or ΔC3 mutant Ad5E4ORF1, as indicated on the left of each panel) were grown on glass cover slips, starved for 4 h, immunostained with anti-HA and anti-VE-cadherin (VE-CAD), as indicated on the top of panels. Cell nuclei were stained with DAPI. The scale bar represents 10 μm. Data shown represent experiments using six HUVEC lines. *B*, coimmunoprecipitation of Ad5E4ORF1 and DLG1 in both the cytosol and the nucleus. Indicated cells were starved for 4 h before lysis. Cell lysates were fractionated into cytosol and nucleus pools and then subjected to anti-HA immunoprecipitation and immunoblot. GAPDH and histone H3 were included as cytosol and nucleus markers, respectively. (*Right panels*) Quantification of Ad5E4ORF1 and DLG1 cytosolic *versus* nuclear distribution from input (cell lysate) blots. Paired *t* test (mean ± SD; n = 3 HUVEC lines). *C*, coaggregation of Ad5E4ORF1 and DLG1 proteins in HUVECs. DLG1 (with insert I3), N terminally fused to ZsGreen and under a tetracycline response element (TRE) promoter, was briefly induced in HUVECs with or without HA-Ad5E4ORF1 coexpression in starving conditions before immunostaining as in (*A*). The scale bar represents 10 μm. Data presented represent experiments with three HUVEC lines. *D*, subcellular localization of PIP3 revealed by a PIP3-binding GFP fusion protein. The AKT1 PHD domain, N terminally fused to GFP and under a TRE promoter, was briefly induced in HUVECs with or without HA-Ad5E4ORF1 in starving medium. The K14A mutant PHD is defective in PIP3 binding. Immunostaining and microscopy were performed as in (*A*). The scale bar represents 10 μm. Data shown represent results from 5 HUVEC lines. *E*, identification of IQGAP1 interaction-specific Ad5E4ORF1 mutations. (*Top panel*) HA immunoprecipitation blot analysis of HUVECs expressing indicated Ad5E4ORF1 alleles, performed as in Fig. 1*D*. (*Lower panels*) Quantification of IQGAP1 and DLG1 IP efficiency. One-way ANOVA with Tukey’s test (mean ± SD; n = 3 HUVEC lines). *F*, Rac1 activation in HUVECs assayed by Rac1-GTP pull-down and immunoblot. (*Right panel*) Quantification of Rac1-GTP (IP) relative to total Rac1 in input. One-way ANOVA with Tukey’s test (mean ± SD; n = 4 HUVEC lines). *G*, random cell migration speed measured by sing-cell tracking. Expressed relative to vector control, which is 44.1 ± 6.5 μm/h (mean ± SD). One-way ANOVA with Tukey’s test (mean ± SD; n = 3 HUVEC lines). *H*, collective cell migration speed measured in gap closure of wounded cell monolayers. Expressed relative to WT Ad5E4ORF1, which is 21.8 ± 6.2 μm/h (mean ± SD). One-way ANOVA with Tukey’s test (mean ± SD; n = 3 HUVEC lines). Ad5E4ORF1, human adenovirus serotype 5 early gene E4ORF1; DAPI, 4′,6-diamidino-2-phenylindole; DLG1, discs large homolg 1; HA, hemagglutinin; HUVEC, human umbilical vein endothelial cell; LDS, lithium dodecyl sulfate; PHD, plant homeodomain; PI3K, phosphatidylinositol 3-kinase; PIP3, phosphatidylinositol 3,4,5-trisphosphate; Rac1, Ras-related C3 botulinum toxin substrate 1; Rac1-GTP, GTP-loaded Rac1.

DLG1 has been proposed to bring E4ORF1, along with its associated PI3K complex, to the plasma membrane, where PI3K substrate PIP2 is enriched (27, 28). To test this hypothesis in human primary cells, we examined Ad5E4ORF1 subcellular localization in HUVEC by indirect immunofluorescence staining (Fig. 2*A*). Anti-HA staining of Ad5E4ORF1 in confluent HUVECs revealed broad distribution in both the nucleus and cytosol, with 39% of cells (±19.1% SD, n = 6 HUVEC lines) showing distinct enrichment at cell-cell contacts, where Ad5E4ORF1 colocalized with VE-cadherin, an endothelial specific junctional adhesion protein (Fig. 2*A*, middle row). No HA signal was detected in vector control cells (top row), confirming the specificity of the E4ORF1 staining. Strikingly, only 1% ± 1.6% of ΔC3 mutant-expressing cells (mean ± SD, n = 6 HUVEC lines) showed significant enrichment at cell-cell contacts (bottom row), supporting a critical role of PDZ-association in E4ORF1 membrane recruitment,

We next employed a cell fractionation approach to confirm Ad5E4ORF1’s broad subcellular distribution and to further examine its interaction with DLG1 across different cellular compartments (Fig. 2*B*). As expected, Ad5E4ORF1 protein was distributed approximately equally between the nucleus and cytosol of endothelial cells (lanes 3, 4; left graph). Notably, while roughly two-thirds of DLG1 proteins resided in the cytosol of naïve HUVECs, a significant portion shifted to the nucleus in the presence of Ad5E4ORF1 (lanes 1–4; right graph). Co-IP experiments revealed a robust Ad5E4ORF1-DLG1 association in both the nuclear and cytoplasmic fractions (lanes 7 and 8), indicating that the two proteins associate in multiple compartments. These findings suggest that, in addition to facilitating Ad5E4ORF1 recruitment to the plasma membrane, DLG1 may also be escorted into the nucleus by E4ORF1, at least under our experimental conditions tested. The nuclear localization of Ad5E4ORF1 is consistent with its previously reported Myc-activating function in immortalized epithelial cells. This suggests that the function may be conserved in primary human cells, and that DLG1 could potentially play a role in this process.

### DLG1 and Ad5E4ORF1 coaggregate throughout the cell

Unlike in epithelial cells, where DLG1 predominantly localizes to cell-cell contacts, DLG1 in endothelial cells has been reported to be primarily cytoskeleton-associated (39). To verify DLG1 localization in HUVECs, we initially performed immunofluorescence staining of endogenous DLG1 but were unable to obtain satisfactory signals, likely due to low DLG1 expression in HUVECs. Therefore, we turned to ectopic expression of fluorescently tagged DLG1 fusion proteins for further analysis.

DLG1 exists as multiple isoforms generated *via* alternative splicing (40). Particularly relevant to its role in E4ORF1-mediated AKT activation are two short insert sequences, I2 and I3, that are alternatively included between the SH3 and GUK domains. Notably, only I3-containing DLG1 isoforms support AKT activation (19). Using molecular cloning from pooled HUVEC complementary DNA, we recovered four DLG1 isoforms, different at two positions (1): the I2/I3 region (either I2 or I3), and (2) a region between L27 and the first PDZ domain, containing either insert 1B alone or inserts 1A and 1B in tandem.

For localization and interaction studies, we selected the DLG1-I1A-I1B-I3 isoform, fused it to ZsGreen at the N terminus, and expressed it under a doxycycline-inducible promoter to maintain low, less toxic expression levels (Fig. 2*C*).

ZsGreen alone localized broadly throughout the cell, with a cytoskeleton-like pattern in the cytosol and dense accumulation in the nucleus (Fig. 2*C*, *top row*). This distribution was not altered by Ad5E4ORF1 coexpression (lower middle row). When fused to DLG1, ZsGreen-DLG1 became predominantly cytosolic, with distinct enrichment at the cell periphery (upper middle row), consistent with the known role of I3 insert in DLG1 membrane targeting (41). Upon coexpression with Ad5E4ORF1, both ZsGreen-DLG1 and E4ORF1 relocalized in all coexpressing cells, colocalizing at cytosolic puncta of varying sizes, while E4ORF1 was notably depleted from the nucleus (bottom row). In addition, DLG1 colocalized with E4ORF1 at cell-cell contacts in most cells. These observations support a potential role for DLG1 in recruiting Ad5E4ROF1 to the plasma membrane.

Further characterization revealed that these cytosolic loci did not colocalize with markers for early endosomes (EEA1; Fig. S1), late endosomes (Rab7; Fig. S2), or focal adhesions (paxillin; Fig. S3). This suggests that the observed puncta are nonmembranous protein aggregates, likely resulting from overexpression of two oligomerization-prone proteins (23, 42). The coaggregation of DLG1 and E4ORF1 reflects a high-affinity interaction that may enhance their local concentrations at specific subcellular sites, thereby amplifying downstream signaling.

### Ad5E4ORF1 activates PI3K at the plasma membrane

Given that PI3K signaling can be activated at the plasma membrane (14), in the cytoplasm (43), and in the nucleus (44), we next monitored PI3K activity by tracking cellular PIP3 levels using a fluorescent PIP3-binding PH domain from AKT1 fused to GFP (PHD-GFP; Fig. 2*D*). Under basal conditions, PHD-GFP localized to both nuclei and cell-cell contacts in HUVECs (top row); in the presence of Ad5E4ORF1 (middle row), PHD-GFP enrichment at cell-cell contacts significantly increased in 81.8% ± 24.7% of cells (n = 5 HUVEC lines), while its nuclear location remained unchanged (middle row). As a negative control, we expressed the K14A mutant PHD-GFP, which is specifically defective in PIP3-binding (45). This mutant retained its normal nuclear distribution but rarely localized to cell-cell contacts, showing enrichment in only 2.5% ± 2.5% of cells (n = 5 HUVEC lines) (bottom row). These observations indicate that the plasma membrane, particularly at cell-cell contacts, is the predominant site of Ad5E4ORF1-stimulated PIP3 synthesis in endothelial cells.

### Ad5E4ORF1 activates Rac1, inhibits cellular motility, and enhances collective cell migration

Cell receptor-mediated activation of PI3K stimulates both the AKT and Rac1 pathways. Given that IQGAP1 is both an effector and a regulator of Rac1 (46, 47, 48), we asked whether Ad5E4ORF1 activates Rac1 and how its recruitment of IQGAP1 might affect the outcome.

We first screened for Ad5E4ORF1 mutations that specifically disrupt its interaction with IQGAP1 (Fig. 2*E*). We found that substitution of E87 with alanine (E87A) or glutamine (E87Q) diminished IQGAP1 co-IP with Ad5E4ORF1 from transduced HUVEC lysate (left graph). Notably, E87A also slightly increased the amount of Ad5E4ORF1-associated DLG1 (right graph), suggesting potential competitive interactions between the two scaffold proteins.

Next, we assessed Rac1 activation by pulling down active, GTP-loaded Rac1 (Rac1-GTP) from HUVEC lysates using Pak1 protein-binding domain-coupled resins, followed by immunoblotting. Compared to vector control cells, expression of Ad5E4ORF1 significantly increased levels of active Rac1. This activation was abolished by the ΔC3 mutation but unaffected by E87Q (Fig. 2*F*), indicating that Ad5E4ORF1 activates Rac1 independently of its interaction with IQGAP1.

Given the well-established roles of Rac1 and IQGAP1 in regulating cytoskeleton dynamics (49), we next investigated the impact of Ad5E4ORF1 on both individual cell motility and collective migration. By tracking motion trajectories of individual HUVECs under starving conditions, we observed that Ad5E4ORF1 reduced random cellular motility by approximately half, an effect requiring Ad5E4ORF1’s PDZ-binding motif but not its interaction with IQGAP1 (Fig. 2*G*). The reduced cell motility may result from the sustained and symmetric distribution of PIP3 across the plasma membrane in Ad5E4ORF1-expressing cells (Fig. 2*D*), potentially disrupting the PIP3 polarization required for directed migration (50). Suppressing cell motility may serve to conserve energy and reduce metabolic demands, thereby enhancing endothelial cell survival under starvation.

To study collective migration, we performed wound healing assays on HUVEC monolayers under starved conditions as previously described (51). We observed that Ad5E4ORF1 increased gap closure speed by approximately fivefold compared to the vector control, while the ΔC3 mutant enhanced closure speed by nearly threefold, though it remained significantly less effective than WT (Fig. 2*H*). The E87Q mutant also appeared considerably less effective than WT in enhancing gap closure, although the difference was not statistically significant. These results, together with Ad5E4ORF1 inhibition of cell random migration (Fig. 2*G*), suggest that Ad5E4ORF1 markedly increases cellular directional persistence, likely through its interactions with both DLG1 and IQGAP1. The reduced cell motility, along with higher collective persistence, would allow for more efficient vessel network formation under starved conditions.

### Ad5E4ORF1 selectively engages the PI3K isoform p110α-p85β

**Figure 3:**
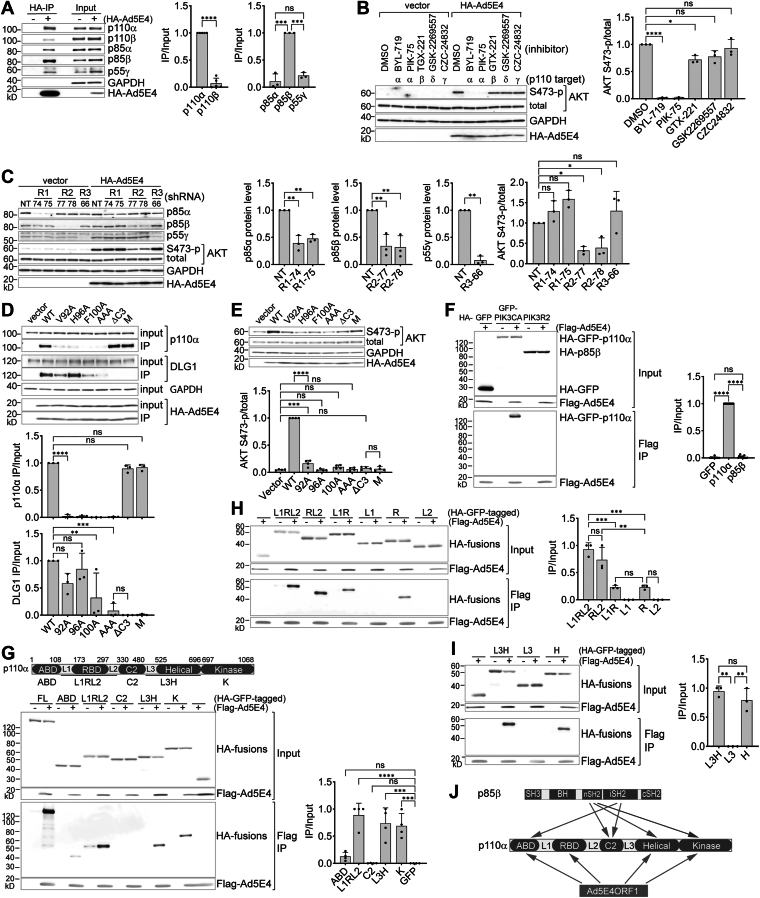
**Ad5E4ORF1 physically and functionally engages PI3K isoform p110α-p85β through multiple-domain interactions**. *A*, coimmunoprecipitation of Ad5E4ORF1 with PI3K proteins. Whole cell lysates (in NP-40 buffer) of vector control (−) and HA-Ad5E4ORF1-expressing (+) HUVECs were subjected to anti-HA immunoprecipitation. HA beads-bound proteins and 0.6% of the corresponding input proteins were analyzed by western blot. (*Right panels*) Relative IP efficiency (IP/Input) of different PI3K subunit isoforms quantified from left immunoblots. Paired *t* test or one-way ANOVA-Tukey’s test (mean ± SD; n = 5 or 3 HUVEC lines). *B*, effect of isoform-specific PI3K inhibitors on Ad5E4ORF1-mediated AKT activation. HUVECs as in (A) were starved for 4 h and then treated with indicated PI3K inhibitors (0.5 μM in DMSO) for 1 h before being lysed into LDS loading buffer for western blot. (*Right panel*) Quantification of AKT phosphorylation in Ad5E4ORF1 cells from left immunoblots. One-way ANOVA-Tukey’s test (mean ± SD; n = 3 HUVEC lines). *C*, effect of shRNA-mediated PI3K regulatory subunit knockdowns on Ad5E4ORF1-mediated AKT activation. Lentiviral shRNAs targeting PIK3R1 (#74, #75), PIK3R2 (#77, #78), or PIK3R3 (#66) were doxycycline-induced in HUVECs for 4 days. After a 4-h starvation, cells were lysed in LDS loading buffer for western blot. (*Right panels*) Quantification of PI3K regulatory subunits knockdown efficiency and AKT phosphorylation in Ad5E4ORF1 cells from left immunoblots. One-way ANOVA-Tukey’s test or paired *t* test (mean ± SD; n = 3 HUVEC lines). *D*, interactions of mutant Ad5E4ORF1 proteins with p110α and DLG1 analyzed by coimmunoprecipitation as in (*A*). (*Lower* panels) Quantification of p110α and DLG1 IP efficiency (normalized to HA-Ad5E4ORF1 in IP) from upper immunoblots. One-way ANOVA-Tukey’s test (mean ± SD; n = 3 HUVEC lines). *E*, western blot analyses of indicated Ad5E4ORF1 mutant cell lysates, performed as in (*B*). M, a single methionine added to Ad5E4ORF1 C terminus. (*Lower panel*) Quantification of AKT phosphorylation from top immunoblots. One-way ANOVA-Tukey’s test (mean ± SD; n = 4 HUVEC lines). *F*, coimmunoprecipitation of Ad5E4ORF1 with p110α. HA-tagged GFP, GFP-p110α fusion, or p85β was coexpressed with Flag-Ad5E4ORF1 (+) or nontagged Ad5E4ORF1 (−) in HEK293T cells. Whole cell lysates (in NP-40 buffer) were subjected to anti-Flag immunoprecipitation; input and beads-bound proteins were analyzed by western blot. (*Right panel*) Quantification of p110α and p85β IP efficiency (normalized to Flag-Ad5E4ORF1 in IP) from left immunoblots. One-way ANOVA-Tukey’s test (mean ± SD; n = 12 independent experiments). *G*, coimmunoprecipitation of Ad5E4ORF1 with p110α fragments. Performed as in (*A*) on cells coexpressing Ad5E4ORF1 and HA-GFP fusions of full-length (FL) or indicated p110α fragments. The upper scheme shows defined p110α domains and linker regions (L1, L2, and L3). (*Right panel*) Quantification of IP efficiency of different p110α fragments from immunoblots on the left. Where nonspecific binding was observed, signal intensity from the control IP was subtracted from that of the Flag-E4ORF1 IP. IP efficiency of each p110α fragment was further normalized to the amount of Flag-Ad5E4ORF1 in the IP. One-way ANOVA-Tukey’s test (mean ± SD; n = 4 experiments). *H* and *I*, fine mapping of Ad5E4ORF1 interactions with p110α L1RL2 and L3H regions by coimmunoprecipitation as in (*G*). Linker regions alone did not bind Ad5E4ORF1 and were used as negative controls in place of HA-GFP. One-way ANOVA-Tukey’s test (mean ± SD; n = 3 experiments). *J*, schematic model of Ad5E4ORF1-PI3K interaction. ABD, adapter p85-binding domain; Ad5E4ORF1, human adenovirus serotype 5 early gene E4ORF1; AKT, protein kinase B; DLG1, discs large homolg 1; DMSO, dimethyl sulfoxide; HA, hemagglutinin; HEK293T, human embryonic kidney 293T; HUVEC, human umbilical vein endothelial cells; IP, immunoprecipitation; LDS, lithium dodecyl sulfate; PI3K, phosphatidylinositol 3-kinase; RBD, RAS-binding domain.

E4ORF1 proteins have previously been reported to physically interact with class IA PI3K both *in vitro* and *in vivo* (28). However, our initial cross-linking Ad5E4ORF1 immunoprecipitation followed by mass spectrometry failed to detect PI3K protein peptides specifically associated with Ad5E4ORF1. We therefore performed non—crosslinking co-IP and immunoblotting in HUVECs to address the issue. Under these conditions, Ad5E4ORF1 specifically co-immunoprecipitated with multiple isoforms of both the regulatory and catalytic subunits of PI3K (Fig. 3*A*). Comparison of IP efficiency (IP/input) indicates that Ad5E4ORF1 selectively binds p110α over p110β (left graph), and p85β over p85α and p55γ (right graph). These findings suggest that Ad5E4ORF1 selectively associates with the p110α-p85β isoform of PI3K.

We next determined the role of p110α-p85β in Ad5E4ORF1 AKT activation by applying class I PI3K isoform-specific inhibitors (Fig. 3*B*) and shRNA-mediated knockdown of regulatory subunits (Fig. 3*C*). Inhibition of p110α, with two specific inhibitors, nearly abolished AKT activation in Ad5E4ORF1 cells, while inhibitors targeting the other isoforms had little or no effect at the same dose (Fig. 3*B*). Moreover, despite similar protein knockdown efficiencies in Ad5E4ORF1 cells (Fig. 3*C*; *left three graphs*), only knockdown of p85β significantly reduced AKT activation (right graph). Notably, AKT activation in naïve HUVECs was not affected by knockdown of any of the three regulatory subunits. Taken together, Ad5E4ORF1 AKT activation specifically requires the catalytic activity of p110α and the presence of p85β.

We next sought to address the role of the Ad5E4ORF1-PI3K physical interaction in Ad5E4ORF1-mediated AKT activation. First, we found that individual alanine mutations of Ad5E4ORF1 amino acids V92, H96, and F100 within the M2 domain (20) markedly reduced its interaction with p110α in co-IP experiments, although mutating all three residues (AAA) showed no additive effect (Fig. 3*D*; *upper graph*). Of note, while F100A and AAA additionally reduced interaction with DLG1, V92A, and H96A largely preserved DLG1 interaction (Fig. 3*D*; *lower graph*). In contrast, alterations of Ad5E4ORF1’s C-terminal PDZ-binding motif, by either deletion (ΔC3) or addition of a methionine (M), abolished its binding to DLG1 without impacting its binding to p110α. Therefore, Ad5E4ORF1 recruits p110α and DLG1 independently through separate and overlapping E4ORF1 domains.

We then investigated how these disrupting mutations affect Ad5E4ORF1 AKT activation (Fig. 3*E*). Although V92A retained low, residual levels of AKT activation, H96A and F100A, as well as the ΔC3 and M mutations, reduced AKT phosphorylation to basal levels, highlighting the essential role of Ad5E4ORF1-associated PI3K in AKT activation.

### Ad5E4ORF1 targets p110α-p85β through interactions with multiple domains of p110α

In the canonical class IA PI3K activation pathway, activated receptors and their adaptor proteins interact with both the catalytic and regulatory subunits to promote catalytic activation and membrane recruitment (14).

However, the influenza A virus nonstructural protein 1 (NS1) protein activates P13K signaling by specifically binding to p85β (52). To determine how Ad5E4ORF1 interacts with the p110α-p85β complex, we first sought to identify which subunit is directly targeted by Ad5E4ORF1. To this end, we coexpressed HA-GFP-p110α (with GFP included to stabilize p110α) or HA-p85β along with either Flag-Ad5E4ORF1 or untagged Ad5E4ORF1 in human embryonic kidney 293T (HEK293T) cells, followed by anti-Flag immunoprecipitation (Fig. 3*F*). Immunoblot analysis of the pull down revealed a specific interaction between Ad5E4ORF1 and GFP-p110α, but not with p85β. As Ad5E4ORF1 did not pull down GFP alone, these results identify p110α as the primary target of Ad5E4ORF1 within the PI3K complex.

We next conducted similar experiments with GFP fusions containing nonoverlapping fragments of p110α to identify the domains involved in Ad5E4ORF1 interactions (Fig. 3*G*). The analysis revealed independent interactions between Ad5E4ORF1 and multiple p110α regions, including L1RL2 [Ras-binding domain (RBD)] plus neighboring L1 and L2 linker regions), L3H (C2-helical linker plus helical domain), and the kinase domain (K). Although not statistically significant, specific binding to adaptor p85-binding domain was consistently observed across all experiments. These independent interactions, though each weaker than with full-length (FL) p110α, may act additively or synergistically in p110α targeting by Ad5E4ORF1.

Further dissection of the L1RL2 region (Fig. 3*H*) revealed that neither linker region alone binds Ad5E4ORF1, while the RBD alone binds reproducibly but at levels that are not statistically significant. However, inclusion of the L2 region, but not L1, markedly enhanced RBD binding to Ad5E4ORF1, suggesting that RBD and L2 bind to Ad5E4ORF1 as a single functional module. Similar analysis of the L3H region revealed that Ad5E4ORF1 interacts primarily with the helical domain, with minimal contribution from the L3 linker region (Fig. 3*I*).

Thus, as depicted in Figure 3*J*, Ad5E4ORF1 selectively targets the p110α-p85β heterodimer through extensive and exclusive contacts with p110α that span the adaptor p85-binding domain, RBD-L2, helical, and kinase domains.

### DLG1 and PI3K recruitment alone does not explain differential AKT activation by E4ORF1 variants

**Figure 4:**
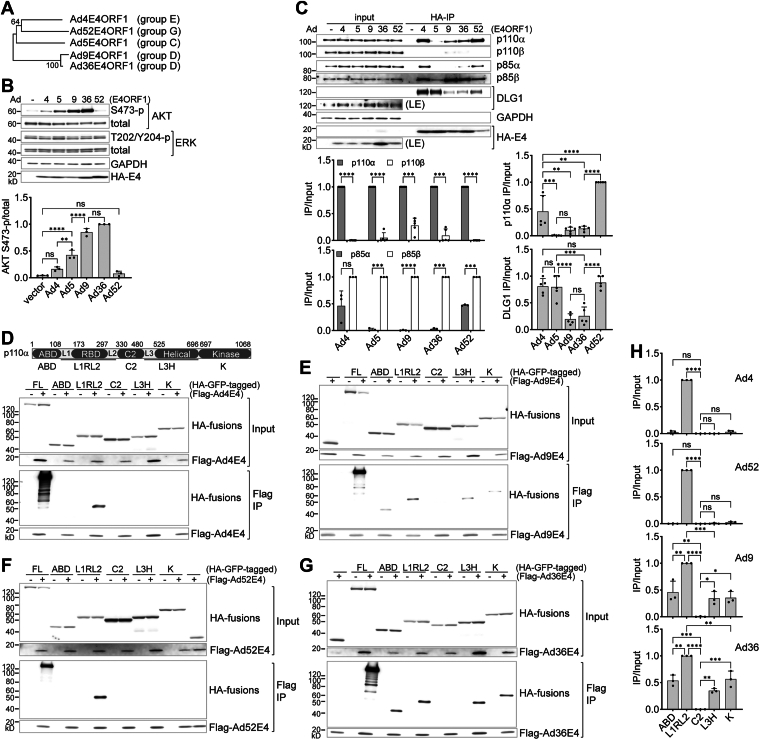
**Multiple-domain interaction with p110α correlates with potent AKT activation by E4ORF1**. *A*, a phylogeny of human adenoviral E4ORF1 proteins of different serotypes, produced from Molecular Evolutionary Genetics Analysis Version 11 using ClustalW alignment and Minimum-evolution Bootstrap. Confidence values shown at applicable branches; B = 500 bootstrap replications. *B*, western blot analyses of HUVECs lentivirally expressing HA-tagged E4ORF1 proteins of human adenoviral serotypes 4, 5, 9, 36, and 52. Cells were starved for 4 h before lysis in LDS loading buffer. (*Lower panel*) Quantification of AKT phosphorylation from upper immunoblots. One-way ANOVA-Tukey’s test (mean ± SD; n = 3 HUVEC lines). *C*, cells as in (*B*) were subjected to anti-HA immunoprecipitation in NP-40 buffer. Beads-bound proteins and 2% of the corresponding input proteins were analyzed by western blot. Longer exposures (LE) of the input blots for DLG1 and HA-E4 were presented. (*Lower left panels*) Relative p1101α/β and p85α/β IP efficiency from individual E4ORF1 serotype HUVEC cells. Paired *t* test (mean ± SD; n = 5 or 3 HUVEC lines). (*Lower right panels*) Relative p110α and DLG1 IP efficiency (normalized to E4ORF1 in IP) across different serotype HUVEC cells. One-way ANOVA-Tukey’s test (mean ± SD; n = 5 or 3 HUVEC lines). *D*, *E*, *F*, and *G*, comparison of p110α domain interactions with Ad4, Ad9, Ad36, and Ad52 E4ORF1 proteins. Coimmunoprecipitation and western blot were performed as in Figure 3 (*G*). Data shown represent three independent experiments. *H* quantification of p110α fragments IP efficiency (normalized to E4ORF1 in IP) from (*D*), (*E*), (*F*), and (*G*). The C2 region of p110α showed no detectable E4ORF1 binding and was used as a negative control in place of HA-GFP. One-way ANOVA-Tukey’s test (mean ± SD; n = 3 experiments). AKT, protein kinase B; DLG1, discs large homolg 1; HA, hemagglutinin; HUVEC, human umbilical vein endothelial cells; IP, immunoprecipitation; LDS, lithium dodecyl sulfate.

To gain further functional insight into Ad5E4ORF1-p110α interactions, we expanded our analysis to include additional natural E4ORF1 variants with varying capacities to activate AKT. We selected E4ORF1 proteins from Ad4, Ad9, Ad36, and Ad52, representing three distinct human adenovirus groups. These variants were chosen in part because they can be stably expressed at comparable levels in HUVECs (Fig. 4*A* and *B*).

Both Ad4 and Ad52 E4ORF1 proteins show minimal AKT activation (Fig. 4*B*). In contrast, Ad9 and Ad39 E4ORF1s, more similar to each other in protein sequence, robustly activate AKT, showing the highest activity among the variants tested. As previously observed for Ad5E4ORF1, none of the tested E4ORF1s activates ERK signaling, confirming their selective engagement of the PI3K-AKT pathway.

To evaluate whether differences in AKT activation correlated with PI3K complex association, we performed anti-HA immunoprecipitation in HUVECs to assess each E4ORF1 variant’s binding to PI3K subunits (Fig. 4*C*).

As observed with Ad5E4ORF1 (Fig. 3*A*), the other E4ORF1 proteins also preferentially associate with p110α overp110β and with p85β over p85α (Fig. 4*C*, *left graphs*). However, the difference in p85β over p85α binding to Ad4E4ORF1 did not reach statistical significance (lower left graph). In addition, Ad52E4ORF1, the other weak activator of AKT, also displayed a greater tolerance for incorporating p85α compared to the more potent variants. These findings suggest that difference in regulatory subunit preference may contribute to the variation in AKT activation potency among E4ORF1 isoforms.

To compare the strength of the p110α interaction with E4ORF1 across variants, we normalized the p110α co-IP efficiency to the amount of E4ORF1 protein recovered in the IP, thereby accounting for differences in E4ORF1 pull-down levels. Although the more active Ad9 and Ad36 E4ORF1s tend to bind p110α more strongly than Ad5E4ORF1, this difference is not statistically significant. In contrast, the least active Ad4 and Ad52 E4ORF1s exhibit the strongest p110α binding (Fig. 4*C*, *upper right graph*). Thus, an E4ORF1’s potential to activate AKT does not strictly correlate with its overall p110α binding strength. Similarly, comparison of DLG1 binding across the E4ORF1 variants using the same approach revealed no strict correlation between DLG1 binding strength and AKT activation. Ad4, Ad5, and Ad52 E4ORF1s bound DLG1 at comparable levels, whereas Ad9 and Ad36 ORF1s exhibited the weakest DLG1 binding (Fig. 4*C*, *lower right graph*). Thus, PI3K and DLG1 recruitment alone cannot fully explain the variability in AKT activation across E4ORF1 variants, indicating the involvement of additional regulatory mechanisms.

### Robust AKT activation is characterized by E4ORF1’s multidomain interaction with p110α

We then examined the nature of the p110α interaction with each E4ORF1 variant by applying the p110α domain-mapping approach described earlier, using anti-Flag-E4ORF1 immunoprecipitation in HEK293T cells (Fig. 4, *D*–*H*). Both Ad4 (Fig. 4*D*) and Ad52 (Fig. 4*F*) E4ORF1s interact exclusively with the L1RL2 region (Fig. 4*H*, upper graphs), and these interactions are considerably weaker than those observed with FL p110α, suggesting that additional p110α domains facilitate E4ORF1-L1RL2 binding. In contrast, Ad9 (Fig. 4*E*) and Ad36 (Fig. 4*G*) E4ORF1s interact with p110α in a manner similar to Ad5E4ORF1, engaging all p110α domains except the C2 domain (Fig. 4*H*, *lower graphs*). This distinction in interaction pattern correlates well with E4OR1 proteins’ AKT activation potential. Thus, potent AKT-activating E4ORF1s are best distinguished by their ability to engage multiple domains of p110α.

## Discussion

We previously established an E4ORF1-based endothelial niche platform for *in vitro* expansion of human hematopoietic stem and progenitor cells (4). Central to this platform is a finely tuned balance between the PI3K-AKT-mTOR and RAS-ERK pathways. The AKT-mTOR pathway promotes endothelial cell survival and stem cell expansion, whereas RAS-ERK signaling drives stem cell differentiation (4). Consistent with our earlier findings on Ad5E4ORF1 (3), all E4ORF1 variants tested in this study selectively activate the PI3K-AKT but not the RAS-ERK pathway in primary human endothelial cells. This contrasts with previous reports of RAS (29) and ERK (30) activation by E4ORF1 in other cell types. Notably, ERK activation by E4ORF1 in the epithelial cells depends on epidermal growth factor receptor (30), which is susceptible to receptor desensitization and cross-inhibition. In addition, PI3K-AKT signaling can suppress ERK activation through well-described negative feedback mechanisms. Thus, E4ORF1-mediated ERK activation is highly context-dependent and intrinsically less robust than its activation of PI3K pathway. Nevertheless, the potential for E4ORF1-induced ERK signaling in endothelium remains an open question and could be explored further to benefit production of differentiated blood cells. Building on our discovery of isoform-specific PI3K activation and the diversity among E4ORF1 variants, our future direction is to engineer stem cell niches that finely control stem cell self-renewal *versus* differentiation.

Cellular scaffold proteins orchestrate signaling networks *via* multivalent protein-protein interactions (53). In this study, we show that Ad5E4ORF1 recruits a group of such scaffold proteins. Specifically, DLG1, in complex with CASK and LIN7C, binds to Ad5E4ORF1 through its C-terminal PBM, while IQGAP1 binds a distinct domain. Consistent with findings from immortalized cell lines (19), we show that DLG1 also facilitates Ad5E4ORF1-mediated AKT activation in human primary endothelial cells. More broadly, DLG1 contributes to AKT activation across multiple receptor stimuli, highlighting its role as a hub of PI3K-AKT signaling. In contrast, IQGAP1, though previously implicated in scaffolding the PI3K-AKT pathway (54), is dispensable for AKT activation by Ad5E4ORF1 in our system. Future studies will further dissect the cellular impact of these E4ORF1-scaffold interactions.

Remarkably, in parallel with its scaffold interactions, Ad5E4ORF1 selectively engages a specific PI3K holoenzyme isoform, p110α-p85β, both physically and functionally. We further demonstrate that this interaction is critical for E4ORF1-induced AKT activation. To our knowledge, this represents the first example of PI3K activation involving a specific holoenzyme isoform. Both the catalytic and regulatory subunits of PI3K can be selectively targeted by cellular or viral proteins. For example, activated GTPases can distinguish among catalytic isoforms: RAS specifically binds the RBD domains of p110α and p110δ; Rac1 and Cdc42 interact with the RBD of p110β (55); and Gβγ targets the linker 3 sequence of p110β (56). For regulatory subunits, recent findings suggest that PI3K activation induced by lysophosphatidylcholine specifically involves p85α through an unknown mechanism (57). In contrast, the influenza NS1 protein selectively binds p85β, thereby preventing the nSH2 domain from inhibiting p110 (52, 58). However, the selective targeting of a PI3K holoenzyme composed of defined catalytic and regulatory subunits has not yet been reported.

To selectively target a specific PI3K holoenzyme isoform, one might assume that both the regulatory and catalytic components must be directly recognized by activated cell receptors or associated adaptor proteins. However, this does not appear to be necessary. Here, we show that Ad5E4ORF1 interacts with multiple domains of p110α, but not with p85β, suggesting that Ad5E4ORF1 may recognize structural features of p110α that are uniquely presented when complexed with p85β, but not with p85α or p55γ. Such isoform-specific structural features of PI3K holoenzymes may enable the development of holoenzyme-specific PI3K inhibitors, offering more precise targeting and improved therapeutic index through reduced off-target toxicity.

Our characterization of p110α interactions with E4ORF1 proteins from several other serotypes of human adenovirus provided further insight into how E4ORF1s target and activate PI3K. The level of E4ORF1-induced AKT activation is not determined by how strong it interacts with DLG1 or p110α alone, but rather by the nature of its interaction with p110α. E4ORF1 proteins from Ad4 and Ad52, which support very low levels of AKT activation, interact solely with RBD of p110α. In contrast, the more potent Ad5, Ad9, and Ad36 E4ORF1 proteins additionally engage three other domains of p110α that are in contact with p85β. These multidomain interactions likely induce global conformational changes in the PI3K holoenzyme, leading to activation of p110α. Moreover, these interactions also appear to influence E4ORF1’s selectivity for p85β. Although Ad4 and Ad52 E4ORF1 proteins show reduced selectivity for p85β and are more permissive to incorporation of p85α (Fig. 4*C*), the broader binding of Ad5, Ad9, and Ad36 E4ORF1s suggests that multidomain engagement of p110α is a key determinant driving p85β specificity.

The advantage of specific targeting of p110α-p85β by E4ORF1 remains unclear, but it may facilitate viral replication across a broad range of cell types. Both p110α and p110β are ubiquitously expressed; however, while p110β is inhibited by the nSH2, iSH2, and cSH2 domains of regulatory subunits, p110α is inhibited only by the nSH2 and iSH2 domains, rendering it more readily activated (59). In addition, p85β is thought to be less inhibitory toward p110 compared to p85α (60). In line with this, the influenza NS1 protein specifically targets p85β to activate PI3K, although its preference for a particular catalytic subunit isoform has not been reported (52, 58). Taken together, these findings suggest that the p110α-p85β holoenzyme may represent an isoform that is more easily activated *via* protein-protein interactions. Alternatively, or perhaps additionally, viruses have evolved to hijack p110α-p85β to exploit an as-yet-unknown isoform-specific signaling that uniquely supports viral replication.

**Figure 5:**
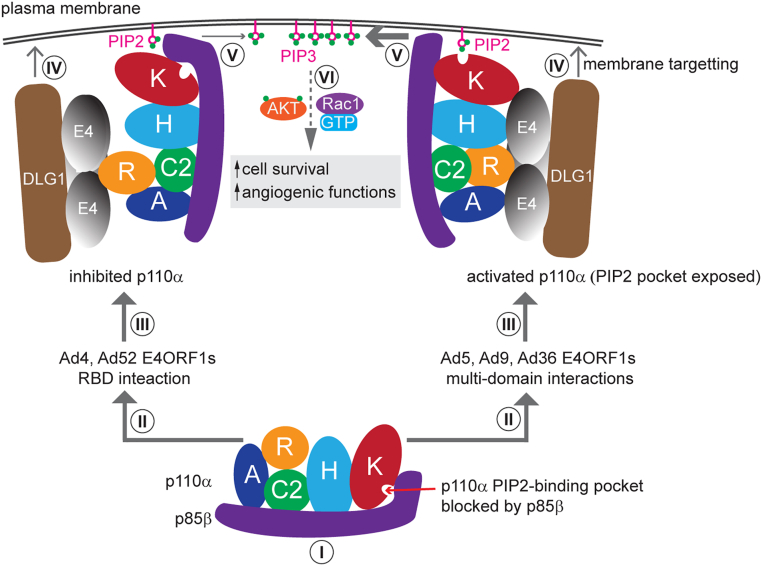
**Mechanistic model of PI3K pathway activation by E4ORF1**. *I*, Within the p110α–p85β complex, p85β (*purple*) inhibits p110α partly by blocking its PIP2-binding pocket. *II*, E4ORF1 proteins selectively bind to p110α when complexed with p85β, exerting bifurcating effects depending on the serotype. *III*, *left arm*: Ad4 and Ad52 E4ORF1 proteins interact only with the Ras-binding domain (RBD) of p110α, causing limited activation due to continued p85β inhibition. Right arm: in contrast, Ad5, Ad9, and Ad36 E4ORF1s engage multiple domains of p110α, effectively relieving p85β inhibition and resulting in robust activation. *IV*, the resulting E4ORF1–PI3K complex is recruited to the plasma membrane *via* DLG1-mediated vesicle trafficking and membrane association. *V*, at the membrane, p110α converts phosphatidylinositol 4,5-bisphosphate (PIP2) into phosphatidylinositol 3,4,5-trisphosphate (PIP3), in proportion to its activation level. *VI*, PIP3 accumulation initiates downstream activation of AKT and Rac1, promoting cell survival and angiocrine signaling. Domains of p110α: A, adaptor-binding domain; R, RAS-binding domain; C2, C2 domain; H, helical domain; K, kinase domain. AKT, protein kinase B; DLG1, discs large homolg 1; PI3K, phosphatidylinositol 3-kinase; Rac1, Ras-related C3 botulinum toxin substrate 1.

Based on our current findings and previous studies (11, 28), we propose a mechanistic model for E4ORF1-mediated PI3K activation (Fig. 5). Class IA PI3K enzymes, including the isoform p110α-p85β, are normally kept inactive in the cytosol by intersubunit inhibition by p85, which includes occlusion of p110α′s PIP2-binding pocket. Trimeric E4ORF1 proteins selectively interact with the p110α-p85β holoenzyme and, *via* their independent interaction with plasma membrane-associated DLG1 (25, 26), recruit PI3K to the membrane in proximity to its substrate, PIP2. The extent of PIP3 synthesis depends on the nature of E4ORF1’s interaction with p110α.

E4ORF1 proteins from Ad4 and Ad52, which bind only to the RBD of p110α, primarily function to recruit catalytically inhibited p110α–p85β to the membrane, resulting in low levels of PIP3 synthesis. In contrast, E4ORF1 proteins from Ad5, Ad9, and Ad36 engage multiple domains of p110α, leading not only to membrane recruitment but also to partial relief of p85β-mediated inhibition. This multidomain engagement results in higher levels of PIP3 synthesis and more robust PI3K pathway.

The extent to which E4ORF1 depends on DLG1 for plasma membrane recruitment, or whether alternative mechanism exists, remains to be fully elucidated. In this study, modest DLG1 knockdown using shRNA led to reduced AKT activation, despite no significant loss of DLG1 from the plasma membrane (data not shown). Given DLG1’s known roles in vesicle trafficking (61) and its interactions with various plasma membrane-associated molecules, including PIP2 (25, 26), it is plausible that DLG1 promotes E4ORF1-mediated PI3K signaling by delivering the E4ORF1-PI3K complex to optimal signaling nanodomains or by repositioning prelocalized, but unproductive, complexes at the membrane. Furthermore, the observed coaggregation of DLG1 and E4ORF1 suggests that DLG1 may facilitate the clustering of PI3K signaling molecules, thereby amplifying signaling output (62).

PI3K signaling is generally wired to respond to physiological extracellular cues through canonical activation mechanisms (14). Currently, no known cellular proteins are capable of autonomously inducing sustained PI3K activation in the manner observed with viral proteins such as E4ORF1 and NS1. However, it is conceivable that plasma membrane-associated proteins such as DLG1 simply require the acquisition of a PI3K interaction to drive sustained signaling. Conversely, cytosolic PI3K-interacting proteins like FOXM1D, which activates p110 by binding both the catalytic and regulatory subunits (63), may acquire the ability to induce sustained PI3K signaling upon gaining plasma membrane localization. Such gain of functions could arise from genetic mutations or through the expression of novel adaptor proteins. We propose that sustained, tonic PI3K activity driven by such noncanonical mechanisms could help maintain certain cellular states, such as quiescence or dormancy, and may underline certain cases of unexplained, cancer-associated PI3K-AKT hyperactivation (64).

In sum, our study uncovered a novel viral strategy for PI3K holoenzyme isoform-specific targeting and activation, offering new avenues for understanding the regulation of endothelial cell survival and homeostatic angiogenic functions. Future structural investigations will be critically required to gain further mechanistic insight into this process. It would be of great interest to know if cellular proteins elicit similar noncanonical PI3K activation in human physiology and pathology.

## Experimental procedures

### Cell culture

HUVECs were isolated from human umbilical cord as described (65) and cultured on gelatin-coated plastic or glass surfaces at 5% O2 in EC growth medium—Medium 199 (Thermo Fisher Scientific 11150–067) supplemented with 15 mM Hepes (Gibco, 15630080), 2 mM glutamine (GlutaMAX, Gibco 35050), 50 μg/ml heparin (Sigma-Aldrich, H3149), 2% fetal bovine serum (FBS), 2.5 ng/ml EGF (PeproTech 100–15), 2.5 ng/ml FGF2 (PeproTech, 100–18B), 2.5 μg/ml insulin (Sigma-Aldrich I9278), 5.0 ng/ml insulin-like growth factor (PeproTech 100–11), 5.0 μg/ml transferrin (PeproTech, 10–366), 5.0 ng/ml sodium selenite (Sigma-Aldrich, S5261), 40.8 μg/ml N-acetyl-L-cysteine (PeproTech, 6169116), 1.0 μg/ml hydrocortisone (Sigma-Aldrich H0888), 0.2% AlbuMax I (Thermo Fisher Scientific, 11020021), 1% (v/v) lipid concentrate (Thermo Fisher Scientific, 11905031), and 0.25% human serum albumin (Grifols, 68516–5216–03).

In some early experiments (Fig. 1, *A*–*G*), ECs were cultured in human endothelial SFM (Thermo Fisher Scientific, 11111044) supplemented with 15 mM Hepes, 2 mM L-glutamine, 50 μg/ml heparin, 2% FBS, and 2.5 ng/ml each of EGF and FGF2.

Other cytokines: HGF (PeproTech 100–39H); SDF1 (R&D Systems 350-NS); and S1P (Sigma-Aldrich 73914). To reduce cytokine effects, cells were starved for 4 h in Medium 199 with 0.1% bovine serum albumin (BSA), 15 mM Hepes, and 2 mM glutamine. For cell survival assays under starvation, cells were kept in minimal medium X-Vivo 20 (Lonza, 180995) for 7 days without medium change.

HEK293T cells (ATCC CRL-11268) were cultured in Dulbecco's modified Eagle's medium (Thermo Fisher Scientific 11965092) supplemented with 10% FBS, 15 mM Hepes, and 2 mM glutamine. Plasmid DNA transfection was performed with Lipofectamine 3000 (Thermo Fisher Scientific L3000015). For transient protein expression, cells were collected 48 h post transfection.

PI3K inhibitors BYL-719 (S2814), PIK-75 (S1205), TGX-221 (S1169), GSK-2269557 (S7937), and CZC-24832 (S7018) were purchased from Selleckchem and dissolved in dimethyl sulfoxide.

### Antibodies

DLG1 (Santa Cruz sc-25661, rabbit; Invitrogen 35–9000, clone 2D11, mouse); IQGAP1 (Santa Cruz sc-10792, rabbit, 1:5000); CASK (Cell Signaling 8968, rabbit); LIN7C (Thermo Fisher Scientific, 51–5600, rabbit); AKT/ERK (Cell Signaling 4685, pan-AKT1, 1:5000; 4060, AKT S473-p, 1:3000; 13038, AKT T308-p; 4695, pan-ERK1/2, 1:5000; 4370, ERK T202/Y204-p, 1:2000. All rabbit); HA tag (Cell Signaling 2367, mouse, 1:2000; 3724, rabbit, 1:2000); Flag tag (GenScript A01868, rabbit, 1:1000–5000); GAPDH (Cell Signaling 5174, rabbit, 1:5000); histone H3 (Abcam ab1791, rabbit, 1:5000); VE-Cadherin (R&D AF938, goat); PI3K p110 (Cell Signaling 4249, p110α, 1:2000; 3011, p110β, 1:2000; both rabbit); p85α (Abcam ab191606, rabbit), p85β (Abcam ab180967, rabbit); p55γ (Proteintech Group 27035-1-P; Cell Signaling 11889; both rabbit); Rab7 (Cell Signaling 95,746, mouse); EEA1 (Cell Signaling 48453, mouse); Paxillin (BD Biosciences 610620, mouse).

Mouse monoclonal antibodies were generated by GenScript against a bacterial recombinant HA-Ad5E4ORF1 fusion protein. Four independent clones were confirmed in immunoblot by reactivity to native Ad5E4ORF1 in cell lysates, but none is suitable for immunostaining or immunoprecipitation.

Unless otherwise indicated, all antibodies were used at 1:1000 for western blotting and 1:200 for immunostaining.

### Protein sample preparation for mass spectrometry analysis

HUVECs expressing GFP or Flag-Ad5E4ORF1 were cultured in three 10-cm dishes to confluence. Medium was replaced with Hanks buffer containing 10 mM cell-permeable protein cross-linker DTBP (Thermo Fisher Scientific, 20665) and incubated at room temperatures for 30 min with slow swirling. Cells were washed once with Hanks buffer and then incubated in Hanks buffer containing 20 mM Tris pH 7.5 for 10 min. Cells were scraped into 0.4 ml radioimmunoprecipitation assay buffer (50 mM Tris–HCl pH 7.5, 150 mM NaCl, 1 mM EDTA, 0.1% SDS, 0.5% deoxycholate, 1% IGEPAL CA-630) supplemented with 1x proteinase and phosphatase inhibitors (Sigma-Aldrich, 5056489001 and 4906837001, respectively). Collected cell lysate was sonicated (Bioruptor, three 30-s pulses at high power setting) and cleared by a 15-min spin at 18,000 g. Protein extracts were incubated with 5 μl anti-FLAG M2 magnetic beads (Sigma-Aldrich, M8823) overnight at 4 °C followed by three washes with radioimmunoprecipitation assay buffer supplemented with 1x proteinase and phosphatase inhibitors at 4 °C. Beads-bound proteins were eluted with 3xFLAG peptide and resolved by reducing SDS-PAGE.

Guided by colloidal blue staining, both GFP and E4ORF1 gel lanes were each excised into eight comparable slices, followed by in-gel trypsin digestion. Enzymatically digested samples were injected onto a C18 ultra performance liquid chromatography reverse phase column and eluted with a linear gradient into an LTQ-Orbitrap MS instrument (Thermo Fisher Scientific). MS data were collected using a dynamic exclusion feature and parallel MS and 6 MS/MS scan events. Collected LC MS/MS data were searched with in-house MASCOT algorithm after spectral data processing using Distiller programs (Matrix Science Lt.).

The proteins present in the GFP and E4ORF1 samples were compiled from those identified in their respective gel slices.

### Westerns immunoblot, protein immunoprecipitation, and active Rac1 pull-down

For whole cell lysate western blot, 12-well confluent cells were directly lysed into 100 μl NuPAGE LDS loading buffer (Thermo Fisher Scientific, NP0007) containing 5% beta-mercaptoethanol aided by sonication (Bioruptor, setting high, 2 × 30 s). Proteins (in 10 μl of lysate) were resolved on 4 to 15% reducing gradient Tris-glycine SDS-PAGE, semidry transferred to nitrocellulose membranes, and incubated with protein-specific antibodies. Horseradish peroxidase-conjugated secondary antibodies (Jackson ImmunoResearch) and the ECL Prime Western Blotting System (GE Healthcare RPN2232) were then used to produce chemiluminescence which was captured with a digital camera (Kindle Biosciences).

Densitometric analysis of protein bands was performed using GelAnalyzer 23.1.1 (www.gelanalyzer.com), developed by Istvan Lazar Jr., PhD and Istvan Lazar Sr., PhD, CSc. Linearity of the raw volume-based quantification method was validated using Coomassie blue-stained SDS-PAGE gels loaded with serial dilutions of BSA. The western blot data presented are representative of at least three independent biological replicates.

For regular protein immunoprecipitation, confluent HUVEC cells from a 10-cm dish or HEK293T cells from two 6-wells were collected by Accutase (Corning 25–058-CI) digestion and lysed in 0.4 ml IGEPAL CA-630 or NP-40 buffer as indicated in Figure legends (50 mM Tris–HCl pH 7.5, 150 mM NaCl, 1% IGEPAL CA-630 (Sigma-Aldrich, I8896) or NP-40 (Thermo Fisher Scientific 28324), 0.2 mM EDTA) facilitated by mild sonication (Bioruptor, setting medium, 2 x 30 s). Cleared lysates were incubated with 8 μl anti-Flag M2 (Sigma-Aldrich, M8823, mouse) or anti-HA (Thermo Fisher Scientific 88836, mouse) magnetic beads for 3 h at 4 °C. Beads were washed three times with lysis buffers and bound proteins eluted in SDS loading buffer. All lysis/wash buffers contain 1x protease and phosphatase inhibitors.

For protein fractionation, cytosolic and nuclear proteins were extracted as described (66). Briefly, HUVECs from a confluent 10-cm dish were first incubated in 0.4 ml hypotonic lysis buffer (10 mM Tris, pH 7.5, 10 mM NaCl, 3 mM MgCl_2_, and 0.3% NP-40) to yield cytosolic fraction. The insoluble fraction was then sonicated into 0.4 ml nuclear lysis buffer (NLB) (20 mM Tris–HCl, pH 7.5, 150 mM NaCl, 3 mM MgCl_2_, 1% NP-40, and 10% glycerol). Following high speed clearing, and prior to immunoprecipitation, cytosolic extract in hypotonic lysis buffer was properly supplemented to match NLB. Beads were washed three times with NLB buffer before elution with lithium dodecyl sulfate loading buffer. All buffers throughout protein fractionation and immunoprecipitation contain 1x proteinase and phosphatase inhibitors.

GTP-loaded Rac1 pull-down was performed using a Rac1 Activation Magnetic Beads Pull-down Assay kit (Millipore 17–10394). Protein lysate of confluent cells from a 100 mm dish was used for each 10 μl Pak1 protein binding domain-coupled resins. In addition, 1x protease and phosphatase inhibitors were present in lysis/wash buffer throughout the experiment.

When input and pull-down (IP) proteins are resolved on the same gel, the amount of input protein is adjusted to avoid signal overexposure.

### Lentiviral plasmid constructs

E4ORF1 proteins encoded by human adenoviruses Ad4 (GenBank: AAT97521.1), Ad5 (AAQ19317.1), Ad9 (CAI05991.1), Ad36 (ACY04495.1), and Ad52 (ABK35065.1) were N terminally tagged with a Flag (DYKDDDDK) or HA (YPYDVPDYA) sequence and expressed under a cytomegalovirus promoter in a pCCL lentiviral vector carrying Blasticidin resistance (31). Infected cells were selected for with 10 μg/ml Blasticidin for 5 days.

HA-tagged PIK3R1 and PIK3R2, and GFP fusions to FL p110α or its fragments were cloned into pCCL between BamHI and SalI restriction sites under a phosphoglycerate kinase (PGK) promoter.

shERWOOD-UltramiR shRNA lentiviral constructs (in pZIP-TRE3G) were purchased from TransOMIC Technologies, and targeted IQGAP1, DLG1, CASK, and LIN7C sequences are as follows: IQGAP1 (ULTRA-3409232, GTCGAAGGTAGATCAGATTCAA; ULTRA-3409233, ACAGCAGCAAGACTACATGAAA; ULTRA-3409234, TGCAGAGAATAATGAATTCATA); DLG1 (ULTRA-3229058, AAACAGTGAAATTCAATTCTAA; ULTRA-3229059, AACGCAGAGTTGAGAAGAAAGA; ULTRA-3229060, AACGTATGTTTAGAAGAAGTTA; all located outside of the I2/I3 coding sequences); CASK (ULTRA-3408028, ATTTGTGACTTGACCACAGCGGC; ULTRA-3408029, TAAAATACGCTTTAGTGTTCCTTT); and LIN7C (ULTRA-3346061, TCTATCAGCAATTCCACCTGGA; ULTRA-3346062, TTAGATTGGAGAGTTTTGTTCT). Infected cells were selected for with 1 μg/ml puromycin for 3 days and shRNA expression induced by 1 μg/ml doxycycline for 4 days.

For inducible expression of Zsgreen fusion proteins, the pZIP-TRE3G vector was modified so that the UltramiR cassette and the Zsgreen stop codon were replaced by a XhoI-EcoRI restriction sequence, giving rise to plasmid TRE3G-XR. DLG1 ORFs were PCR amplified from HUVEC complementary DNAs (forward primer: ACACCTCGAGATGCCGGTCCGGAAGCAAGATACCC; reverse primer, ACACACGCGTTTATAGCTTTTCTTTTGCCGGAACC) and cloned into TRE3G-XR using the XhoI and MluI restriction sites. Four DLG1 isoforms were obtained differing in inserts 1A, 1B, 2, and 3—namely isoforms I1A-I1B-I2, I1A-I1B-I3, I1B-I2, and I1B-I3.

For inducible expression of AKT1-PHD-GFP fusions, a cassette of human AKT1 PH domain (aa1-148) fused to GFP (spaced by 6x glycines) was cloned into pZIP-TRE3G between BamHI and MluI sites, replacing ZsGreen and the downstream UltramiR cassette. A *K14A* mutation was made in PHD to specifically disable PIP3 binding (45).

### Lentivirus production, titration, and VCN determination

All lentiviral vector plasmid DNAs were prepared with DNA Midiprep kit (Qiagen 12145). Viruses were packaged in HEK293T cells by cotransfection with second- or third-generation packaging plasmids. Culture media were collected 48 h post transfection and virus particles concentrated using Lenti-X concentrator (Katara, 631232), resuspended in PBS without magnesium (Corning 21040CV), and stored at −80 °C in small aliquots. Virus titers were determined with Lenti-X p24 titer kit (Katara, 632200).

For viral infection, proper amounts of viruses were applied to proliferating cells to aim for ∼70% infection rate estimated from acquired drug resistance. For VCN determination, genomic DNAs were extracted from cells at least 7 days post infection and quantified by quantitative PCR for a PGK promoter sequence (primers ACGTCTCACTAGTACCCTCGCAGAC; GCTATTGGCCACAGCCCATC) and a lentiviral vector backbone sequence (primers TAGCGGTTTGACTCACGGGGATTTC; GTCAATGGGGCGGAGTTGTTACGAC). DNA standards were prepared from serial dilutions of a PGK promoter-containing pCCL lentiviral vector DNA. VCNs were calculated from genomic vector backbone/PGK ratios by assuming two copies of PGK per diploid genome.

### Fluorescence microscopy analysis

Cells cultured on gelatin-coated glass coverslips (Thermo Fisher Scientific 1254580) placed in 24-wells were fixed in PBS containing 4% paraformaldehyde for 15 min at room temperatures, permeabilized for 20 min in PBS containing 0.1% Triton X-100, and blocked for 1 h in PBS containing 3% BSA and 10% horse serum. Cells were incubated with primary antibodies followed by secondary antibodies conjugated with Alexa 647, 594, 555, or 488. Samples were stained with 4′,6-diamidino-2-phenylindole, mounted in ProLong Gold Antifade Mountant (Thermo Fisher Scientific, P36930), and analyzed by LSM-710 (Carl Zeiss) confocal laser scanning microscopy.

### Cell motility assay

Cells were seeded into 6-well plates (25,000 cells/well) in EC growth medium, allowed to attach for over an hour, serum starved in X-VIVO supplemented with 10 μl/ml EC growth media for 3 h, and then placed into the incubation chamber of a Zeiss Axio Observer Z1 microscope and maintained under normal culture conditions. Cell images were captured at 3-min intervals for 6 h and processed with FIJI plugin TrackMate (67) to obtain the movement speed of about 1000 cells in the field.

### Wound healing assay

Cells were seeded into 6-well plates (200,000 cells/well) in EC growth medium and incubated overnight to ensure high confluence. After a 3-h starvation in X-vivo 20, the monolayers were scratched, washed with PBS, and incubated with fresh X-vivo 20. The plate was placed into the heated incubation chamber of a Zeiss Axio Observer Z1 microscope with motorized stage under cell culture temperature and air concentrations. Time lapse imaging was conducted at 3-min intervals for 12 to 48 h. The wound distance d(t) in at least five places along the boundary was manually measured at 30-min intervals with ImageJ. The gap closing speed was quantified through linear regression $d\left(t_{i}\right)=v_{gap}\cdot t_{i}+d_{0}$ (51).

### Statistics

To account for experimental variation, values were normalized either to a specified control sample or to the highest value within each experiment. Depending on the comparison, either repeated measures one-way ANOVA (two-sided), followed by *post hoc* Tukey’s test with multiplicity-adjusted *p* values, or a paired *t* test (two-tailed) was used. All analyses were performed using GraphPad Prism 10. *p* value notation: ns = not significant; ∗ = *p* < 0.05; ∗∗ = *p* < 0.01; ∗∗∗ = *p* < 0.001; ∗∗∗∗ = *p* < 0.0001.

## Data availability

All data are available within the article and Supporting information files. The mass spectrometry proteomics data have been deposited to the ProteomeXchange Consortium *via* the PRIDE partner repository with the dataset identifier PXD069348.

## Supporting information

This article contains supporting information.

## Conflict of interest

MG is an employee, and SR an unpaid founder of Angiocrine Biosciences, San Diego, CA, USA. All other authors declare that they have no conflicts of interest with the contents of this article.
