# Supplementary material for: Adenovirus E4ORF1 activates isoform-specific phosphatidylinositol 3-kinase signaling in human endothelial cells
Source: J Biol Chem. 2025 Nov 13;302(1):110947. doi: 10.1016/j.jbc.2025.110947 (PMC12743444; doi:10.1016/j.jbc.2025.110947)
Supplement: Supporting Data 2 [file mmc2.pdf]

Supporting Information

Adenovirus E4ORF1 activates isoform-specific phosphatidylinositol 3-kinase signaling in human endothelial cells

Fuqiang Geng[1*], Mariko Kobayashi[1], Yang Lin[1], Jesus Maria Gomez-Salinero[1], Dominick Romano[2], Jean Kanyo[3,4], Jennifer Geng[1], Ying Liu[1], Michael Ginsberg[5], Jae-Hung Shieh[1], Kevin Chen[1], TuKiet T. Lam[3,4], Arash Rafii[1,6], Sina Y Rabbany[1,2], Raphaël Lis[1], and Shahin Rafii[1*]

Table S1. List of proteins identified by mass spectrometry in anti-Flag pulldowns from GFP- and Flag-Ad5E4ORF1-expressing HUVECs (submitted as separate Excel file).

Fig. S1. Overexpressed Ad5E4ORF1 and DLG1 proteins do not colocalize with EEA1 in HUVECs.

Fig. S2. Overexpressed Ad5E4ORF1 and DLG1 proteins do not colocalize with Rab7 in HUVECs.

Fig. S3. Overexpressed Ad5E4ORF1 and DLG1 proteins do not colocalize with Paxillin in HUVECs.

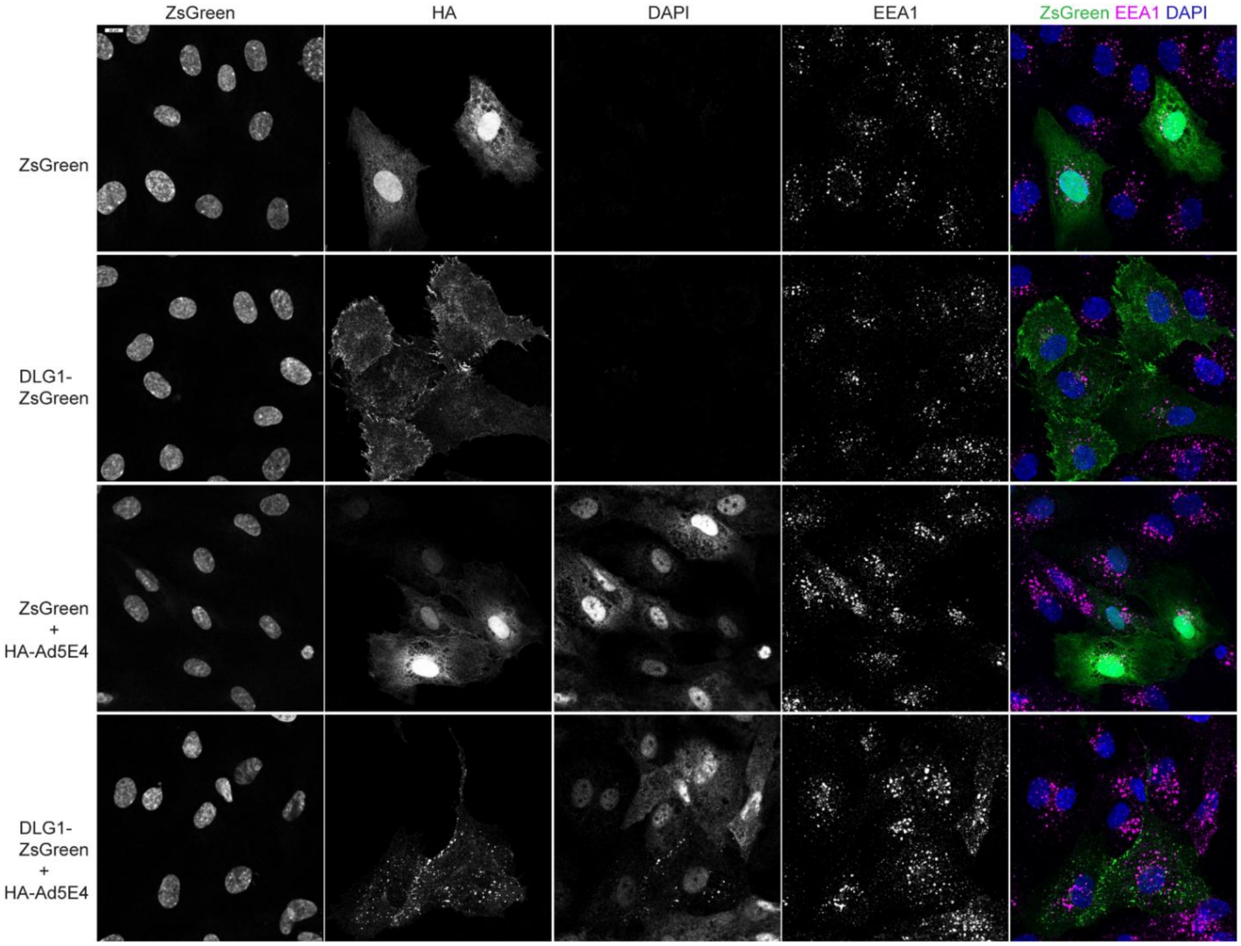

Fig. S1. Overexpressed Ad5E4ORF1 and DLG1 proteins do not colocalize with EEA1 in HUVECs. HUVECs were transfected to express either DLG1-ZsGreen or ZsGreen alone under a TRE promoter, with or without co-expression of HA-Ad5E4ORF1, as indicated on the left of each panel. After brief induction, cells were starved for 4 hours, immunostained for the HA tag and EEA1 (as indicated at the top of the panels), and analyzed by confocal microscopy. Scale bar: 10 μm. The data shown are representative of three independent HUVEC lines.

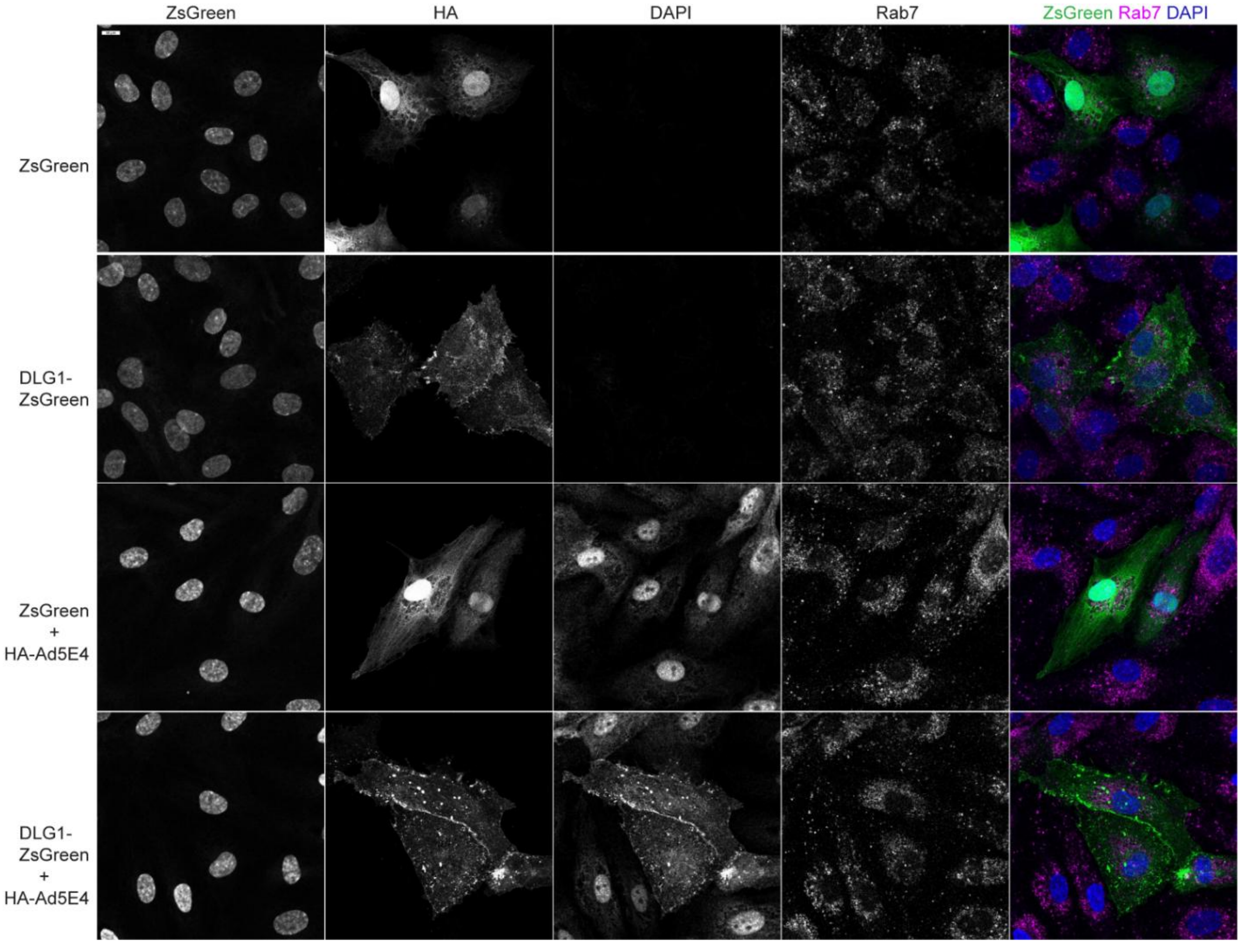

Fig. S2. Overexpressed Ad5E4ORF1 and DLG1 proteins do not colocalize with Rab7 in HUVECs. Confocal analysis as in Fig. S1, using a Rab7 antibody. Scale bar: 10 µm.

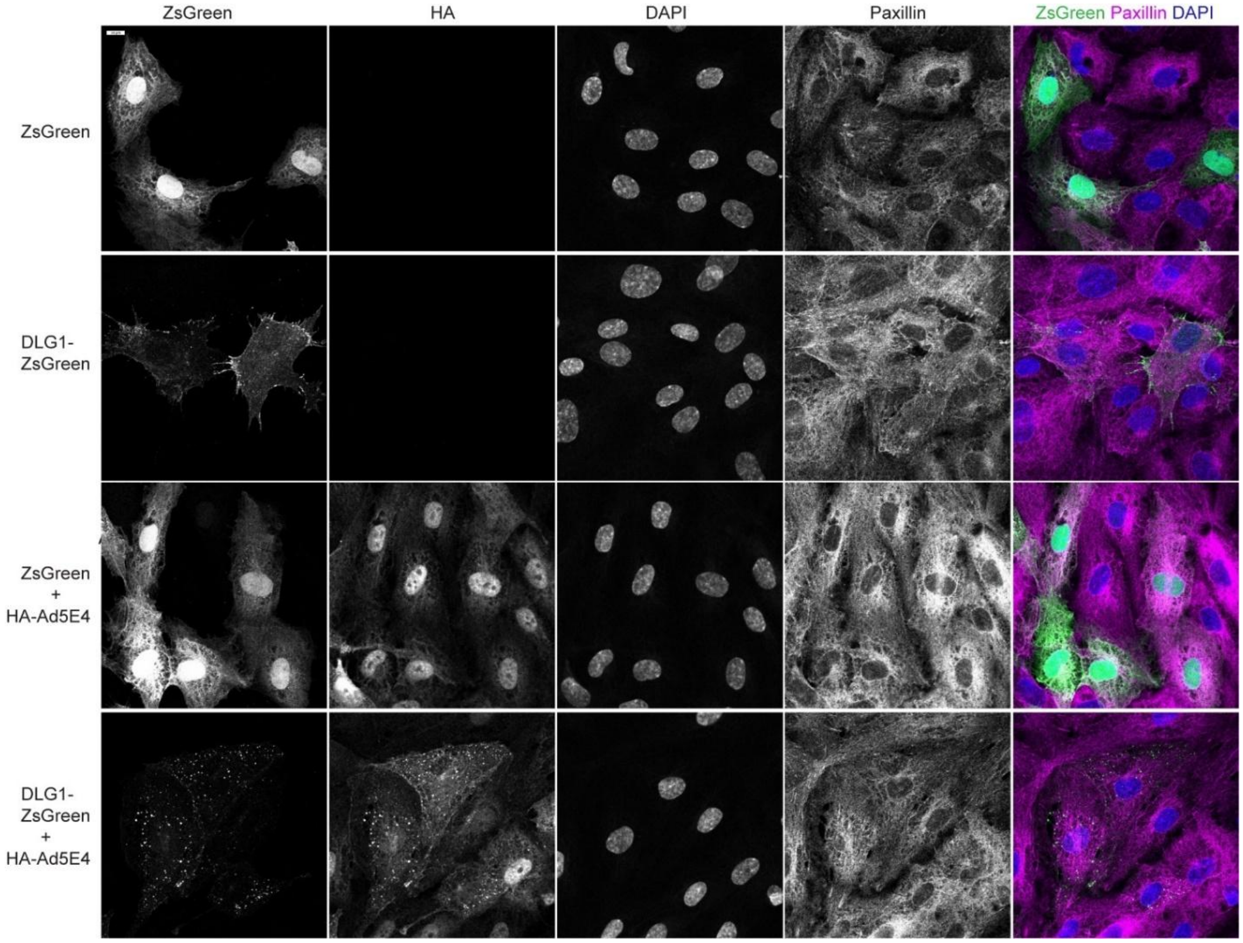

Fig. S3. Overexpressed Ad5E4ORF1 and DLG1 proteins do not colocalize with paxillin in HUVECs. Confocal analysis as in Fig. S1, using a paxillin antibody. Scale bar: 10 μm.
